# Development of the Lupus Interactive Navigator as an Empowering Web-Based eHealth Tool to Facilitate Lupus Management: Users Perspectives on Usability and Acceptability

**DOI:** 10.2196/resprot.4219

**Published:** 2016-05-30

**Authors:** Carolyn Neville, Deborah Da Costa, Murray Rochon, Christine A Peschken, Christian A Pineau, Sasha Bernatsky, Stephanie Keeling, Antonio Avina-Zubieta, Elizabeth Lye, Davy Eng, Paul R Fortin

**Affiliations:** ^1^Division of Clinical EpidemiologyDepartment of MedicineMcGill University Health CentreMontreal, QCCanada; ^2^Jack Digital Productions, IncToronto, ONCanada; ^3^Division of RheumatologyDepartment of MedicineUniversity of ManitobaWinnipeg, MBCanada; ^4^Division of RheumatologyMcGill University Health CentreMontreal, QCCanada; ^5^Division of RheumatologyDepartment of MedicineUniversity of AlbertaEdmonton, ABCanada; ^6^Arthritis Research Centre of CanadaVancouver, BCCanada; ^7^Division of RheumatologyDepartment of MedicineUniversity of British ColumbiaVancouver, BCCanada; ^8^Axe Maladies Infectieuses et ImmunitairesCentre de recherche du CHU de Québec - Université LavalQuebec City, QCCanada; ^9^Division of RheumatologyDepartment of MedicineCHU de Québec - Université LavalQuebec City, QCCanada

**Keywords:** Systemic lupus erythematosus, web-based eHealth tool, self-management, empowerment, usability, navigation

## Abstract

**Background:**

Systemic Lupus Erythematosus (SLE) is a serious, complex, and chronic illness. Similar to most other chronic illness states, there is great interest in helping persons with SLE engage in their disease management.

**Objective:**

The objectives of this study were to (1) develop the Lupus Interactive Navigator (LIN), a web-based self-management program for persons with SLE, and (2) test the LIN for usability and acceptability.

**Methods:**

The LIN development platform was based on the results of preliminary comprehensive needs assessments and adapted from the Oncology Interactive Navigator, a web-based tool developed for persons with cancer. Medical researchers, writers, designers, and programmers worked with clinical experts and persons with SLE to develop content for the LIN. Usability and acceptability of the LIN was tested on individuals with SLE meeting American College of Rheumatology criteria, who were recruited from five Canadian SLE clinics. Participants were provided with access to the LIN and were asked to use it over a two-week period. Following the testing period, participants were contacted for a 30-minute telephone interview to assess usability and acceptability.

**Results:**

The content for the LIN was subdivided into six primary information topics with interview videos featuring rheumatologists, allied health professionals, and persons with SLE. Usability and acceptability of the LIN was tested on 43 females with SLE. Of these, 37 (86%) completed telephone interviews. The average age was 43.6 (SD 15.9) years and disease duration averaged 14.1 (SD 10.8) years. Median time spent on LIN was 16.3 (interquartile range [IQR]:13.7, 53.5) minutes and median number of sessions was 2 (IQR: 1, 3). Overall, Likert ratings (0=strongly disagree; 7=strongly agree) of website usability and content were very high, with 75% scoring >6 out of 7 on all items. All participants agreed that LIN was easy to use, would recommend it to others with SLE, and would refer to it for future questions about SLE. Very high ratings were also given to relevancy, credibility, and usefulness of the information provided. Overall, 73% of the participants rated all topics helpful to very helpful. Participants who reported more prior knowledge about SLE rated items regarding improvement in knowledge and helpfulness relatively lower than persons with less prior knowledge. Most participants commented that the LIN would be very useful to those newly diagnosed with SLE. Minor revisions were recommended.

**Conclusions:**

This study furthers the understanding of the needs in the SLE community and delivers a unique eHealth tool to promote self-management in persons with SLE. The LIN was found to be highly acceptable in content and usability. The information provided on LIN may be most helpful for individuals with less experience with the disease, such as those newly diagnosed, indicating the need to tailor the content for persons with more SLE experience.

## Introduction

Systemic lupus erythematosus (SLE) is a chronic autoimmune disease associated with multi-organ involvement and characterized by frequent flares. The unpredictable nature of the illness and complexity of treatment create serious challenges in disease management to persons with SLE and their health care providers [1]. Self-management interventions are essential to meet these challenges and they must be built on sound person-centered approaches and aimed at self-empowerment strategies. In chronic illness management there is an ever increasing need for patients to play more active roles in their health care, and work in partnership with their health care providers. However, for this to occur, patients need to be equipped with a technology that will provide them with appropriate information and accessible support tools. We used qualitative research methods to better define the information and support needs of persons with SLE and health care providers [2,3]. Oncology Interactive Navigator (OIN) is a web-based tool developed to build a sense of competence around living with cancer, support autonomy, and engage patients and families as partners in care. Based on the OIN and preliminary work in SLE, we developed the Lupus Interactive Navigator (LIN). The LIN was designed to provide education and to support self-management in persons with SLE. This paper reports on the development of the LIN and the results of the testing of usability and acceptability of the LIN.

## Methods

### Development of the Lupus Interactive Navigator

The results of the needs assessments were organized into topic groups to form the basis of the *Table of Contents* and guide content development of the LIN. By adapting the OIN platform and approach, medical researchers, writers, designers and programmers worked with clinical experts and persons with SLE to outline the *Table of Contents* and write the first plain-language draft for the LIN. Required graphics and interview videos featuring rheumatologists, allied health professionals, and persons with SLE were produced to complement the written content.

The content for the LIN was subdivided into six primary information topics that were derived from the qualitative analysis of the results of our focus groups and surveys [2,3]. Each primary topic was subdivided with pull-down tabs for further elaboration on each topic (Table 1).

**Table 1:** Summary of LIN *Table of Contents*.

About Lupus	Symptom Management & Treatments	Accessing Healthcare	Support Services	Family, Friends & Work	Living Well With Lupus
What is Lupus	Symptoms of Lupus	Your Care Team	Community Services search tool to locate available lupus- support resources in close proximity to the user’s postal code	Family & Lupus	Managing Stress & Fatigue
What Causes Lupus?	Medications to Treat Lupus	Communicating with Your Care Team		Lupus & Work	Getting Enough Sleep
Lupus & the Immune System	Side Effects of Medications	Covering Medical Costs		Supporting Someone With Lupus	The Importance of Exercise
A Lupus Diagnosis	Preventing and Managing Flares	Transitioning From Pediatric to Adult Care		Resources for Family Members & Friends	Depression
Prognosis	Monitoring Your Lupus	Accessing Care in Rural Areas			Overcoming the Emotional Hurdles of Lupus
Flares and Remissions	Complementary Therapies				Maintaining a Healthy Diet
Lupus & The Body	Other Therapies				Vaccinations
FAQs/ Myths	Clinical Trials				Pregnancy & Lupus
					Young People & Lupus

### Usability and Acceptability of the Lupus Interactive Navigator

#### Participants

Individuals meeting the 1997 SLE American College of Rheumatology criteria [4] were recruited from five Canadian SLE clinics based in university health centers in Vancouver, Edmonton, Winnipeg, Montreal and Quebec City. Each center had obtained prior approval from their local research ethics board for this study. Consenting participants were provided information on how to access the LIN website and were asked to use their devices (computers, tablets, or mobile phones) to browse LIN over a two-week period. The number of log-ins and the duration of each session were recorded for each participant. Following the two-week testing period, participants were contacted for a 30-minute telephone interview to assess their opinions about the LIN and to identify areas for improvement.

#### Telephone Interview

Content for the telephone interview was established following discussions with members of an expert panel including a rheumatologist, a psychologist, a nurse, the developer of the OIN, and an individual with SLE. The telephone interview consisted of a four-page document including (1) a script to be used as the introduction to each interview, (2) questions to assess demographics and participant characteristics, (3) Likert scales to rate website usability, content, and perceived helpfulness, and (4) open-ended questions to assess overall acceptability and usability of the LIN and to provide recommendations and comments.

*Participant characteristics* included age, marital status, education, disease duration, and factors related to computer usage, including ease with using computers, time spent on the Internet, time spent searching for health information, and type of device used.

#### Website Usability and Content

Likert scales were used to assess website usability and content quality and quantity. Participants were asked to rate 17 items on a 7-point Likert scale (0=strongly disagree; 7=strongly agree). Participants who scored items <3 were asked to elaborate. Scores equal to or >5 were considered moderate to strong agreements. Scores equal to or <4 were considered low to no agreement.

#### Helpfulness

Participants were asked to rate each of the six topic sections using a 5-point Likert scale in terms of how helpful each section was to them (1=not at all helpful; 5=most helpful). Scores equal to or >4 were considered *helpful* to *very helpful*. Scores equal to or <3 were considered low to *not at all helpful*.

#### Recommendations and Comments

Four open-ended questions were asked to further assess if there was information missing, and give participants the opportunity to comment on the overall experience and provide recommendations for improvement.

#### Statistical Analyses

The data was transferred from the interview questionnaire to Microsoft Excel (2007). Means, medians, and percentages were calculated for continuous variables and percentages were calculated for categorical variables.

## Results

### Development of the Lupus Interactive Navigator

**Figure 1:**
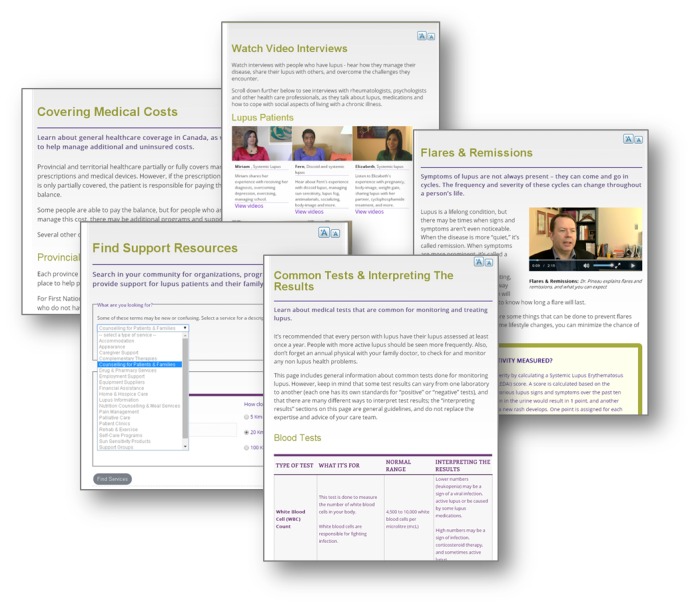
Examples of LIN web pages.

The LIN was constructed from a *Table of Contents* that was developed from the results of the prior focus group studies and surveys performed in preparation for this project [2,3]. An abbreviated *Table of Contents* is provided in Table 1, reflecting the topics that were prioritized by the focus groups and surveys. Figure 1 provides a glimpse of some of the web-based pages that persons with SLE can access once logged into the LIN.

### Usability and Acceptability of the Lupus Interactive Navigator

Forty-three women with SLE were recruited. Of these, six participants did not complete the two-week follow-up telephone interview (time constraints, 2; illness, 2; could not be contacted, 2). Thirty-seven (86%) completed the telephone interviews. Median time spent on LIN was 16.3 (interquartile range [IQR]:13.7, 53.5) minutes and median number of sessions was 2 (IQR: 1, 3).

#### Characteristics of Participants

Table 2 describes the characteristics of the study participants. The average age was 43.6 (SD 15.9) years, disease duration averaged 14.1 (SD 10.8) years, 57% (21/37) were married, and 86% (32/37) had completed post-secondary education. Weekly reported average Internet usage was 15.8 (SD 24.6) hours with 2.8 (SD 0.9) hours used for health information. Ratings for overall experience, website usability, and content are shown in Table 3.

**Table 2:** Participant characteristics (N=37).

	Mean (SD)	n (%)
Age (years)	43.6 (15.9)	
Disease duration (years)	14.1 (10.8)	
Gender (% female)		37 (100)
Marital status (% married)		21 (57)
Education (% post-secondary)		32 (86)
Comfort with computers (% comfortable to very comfortable)		27 (73)
Internet usage per week (hours)	15.8 (24.6)	
Health information searches per week (hours)	2.8 (0.9)	
*Preference for web searching*		
Personal Computer		24 (65)
Tablet		7 (19)
Mobile Phone		6 (16)

**Table 3:** Ratings of website usability and content based on 37 participants (1=strongly disagree; 7=strongly agree).

Statement	Mean (SD)	% equal to or >5
*Overall experience*		
I would recommend this website to others seeking information about lupus	6.8 (0.5)	100
Would go to this website if I had a question about lupus	6.2 (1.5)	89
My friends and family would benefit by accessing this website	6.0 (1.8)	89
*Website design*		
Easy to learn how to use the website	6.7 (0.6)	100
Easy to find information on this website	6.6 (0.6)	97
Easy to read information	6.6 (0.7)	100
Easy to use the website	6.6 (0.6)	100
*Content*		
Information was credible	6.6 (0.8)	97
Information was relevant	6.6 (0.7)	97
Information was useful	6.5 (1.1)	97
Satisfied with the amount of information	5.9 (1.6)	87
Improved knowledge about lupus	5.8 (1.6)	87
Improved knowledge about coping	5.4 (1.9)	78
Improved knowledge about resources	5.4 (1.8)	77
Will help me maintain better health habits	5.3 (1.7)	69
Improved knowledge about medications	5.0 (1.8)	68
Will be useful to help me prepare for next doctor’s visit	5.0 (1.9)	67

#### Overall Experience

All participants strongly agreed that they would recommend this website to other persons with SLE seeking information about SLE and 89% (33/37) would refer to it to answer their own future questions about SLE. Participants also agreed that family and friends would benefit from accessing the LIN.

#### Website Usability

All items assessing website usability received *high* to *very high* ratings. All participants were in *high* agreement that the website was easy to learn and use.

#### Content

Over 97% (36/37) of the participants were in *high* to *very high* agreement that the content was useful, credible, and relevant. Additionally, 86% (32/37) of participants were satisfied with the amount of information provided. Ratings for items relating to gains in knowledge about lupus, coping, and resources were *high* (77-87%). Somewhat lower ratings were given for items relating to gains in knowledge about medications, helpfulness in maintaining good health habits, and preparing for clinic visits (67-69%). Participants stated that the reason for giving lower ratings was prior knowledge about these items. Participants with disease duration <5 years had similar ratings for these items as those with disease duration >5 years. However, there were only 11 participants with short disease duration in our sample.

#### Helpfulness

Table 4 shows the ratings of helpfulness of each of the six major information topics provided in LIN. Overall, 73% (27/37) of the participants rated all topics *helpful* to *very helpful*.

Ratings of perceived helpfulness for each individual major topic varied across topics. The topic *About Lupus* (providing general information about SLE) was perceived as the most helpful (91%) and the topic *Support Services* (providing information about available resources) was considered least helpful (57%). Once again, lower ratings were given by those who reported prior knowledge about SLE than those with less prior knowledge (62% versus 86%).

All participants voiced enthusiasm about LIN and were eager to offer comments and recommendations to further improve this website. None of the participants reported any information missing from the content. However, 26 participants would have preferred more information about specific topics. Of these, the most frequently requested were: more information regarding current research about new medications for SLE (n=9); coping strategies including yoga, meditation, psychosocial, and complementary/alternative treatments (n=9); and adding more support resources on the resource locator for Manitoba and Alberta, including support groups and social workers (n=7).

Minor changes were recommended to improve appearance and usability of the LIN, including changes to font and facilitating drop-down menus. Recommendations to improve content included (1) more videos of physicians and youths, (2) more pictures of rashes and medications, (3) the addition of social networking tools such as a chat room or forum, (4) updates on research and new medications using podcasts, tweets, newsletters, or message boards, and (5) providing a link to show the source of the information provided in the LIN. These adaptations are being implemented.

Overall, the comments were very positive. The most frequent comment was that this website would be most useful to those newly diagnosed with SLE (n=10). Many participants spoke of looking forward to the completed version of LIN that would include a forum for discussion.

**Table 4:** Ratings of helpfulness of the LIN content across information topics based on 37 participants (1=not at all helpful; 5=most helpful).

Section topic	Median (IQR)	% equal to or >4
About Lupus	5 (4,5)	91
Friends, family, and work	5 (4,5)	76
Symptom management and treatment	5 (3,5)	72
Living well with Lupus	5 (3,5)	71
Accessing health care	4 (3,5)	68
Support services	4 (3,4)	57

## Discussion

Tailored web-based programs are becoming increasingly considered as a means of empowering individuals with chronic conditions with the tools and strategies needed to promote self-management. Our study furthers the understanding of needs in the SLE community, and allowed us to develop a web-based tool to build confidence, support autonomy, and empower persons with SLE toward self-management. As SLE is an uncommon yet important disease, this is also an important tool to support health providers caring for people with SLE.

There are a number of potential limitations of our work. First, the study participants were recruited from tertiary care centers and tended to be well educated, and thus may not be entirely representative of the full spectrum of persons with SLE. Second, participants were all females and we cannot generalize these findings to males, who represent 10% of the SLE population. Third, most participants had SLE for several years and their analysis of the LIN was affected by their own experience of living with SLE for that period of time. Many participants mentioned the usefulness of the LIN for persons newly diagnosed with SLE. Several participants also perceived that the LIN would have been most useful at the time of their diagnosis, although they agreed that they still learned from the LIN and would continue to use it to answer questions in the future. This suggests that the LIN could provide different points of entry based on disease duration and experience. Lastly, accessibility of a web-based tool may be suboptimal in some geographic, demographic, or socio-economic groups. However, the LIN can be used from any mobile device such as a tablet or mobile phone.

We assessed the acceptability and usefulness of the LIN as a tool to improve empowerment and self-management in persons with SLE. These results support the value of using a multi-method design that included surveys, focus groups, an expert panel, and interviews when developing programs tailored to specific populations. It is important to acknowledge that tools such as the LIN require ongoing updates and development to respond to new information and user feedback. Other sections which we are already considering for further development include a personalized *SLE Tool Box* which will offer links to an electronic SLE *Health Passport* (personal profile to monitor aspects of health, such as blood pressure), printable information sheets, links to clinical trials, and a forum for discussions relating to SLE.

All participants agreed that the website was easy to navigate and functioned well. The quality and quantity of content were also rated highly. No topics were reported to be missing. This suggests the value of involving patients in the design phase by identifying their needs and preferences when developing eHealth websites tailored to specific populations. Nine participants suggested adding more information about research on new medications for SLE and six participants asked for more information about yoga and meditation as coping strategies. To address these needs, more information will be provided about ongoing research on SLE, and coping strategies delivered interactively via webinars with psychosocial experts are being considered.

### Conclusion

As chronic disease models of care evolve toward self-management, it is increasingly important to develop and validate tools that support providers and engage patients. The LIN is an example of such a tool. It was very well received by patients, and considered easy to navigate with sufficient quantity and quality of content. The information provided on the LIN may be most helpful for individuals lacking experience with the disease, such as those newly diagnosed. Our results suggest the need to tailor the content for persons with more SLE experience.

## Abbreviations

CIHR: Canadian Institutes of Health Research
GSK: GlaxoSmithKline
IQR: interquartile range
JDP: Jack Digital Productions
LIN: Lupus Interactive Navigator
OIN: Oncology Interactive Navigator
PSHI: Partnerships for Health System Improvement
SLE: Systemic lupus erythematosus
